# Regional variations in and correlates of disability-free life expectancy among older adults in China

**DOI:** 10.1186/1471-2458-10-446

**Published:** 2010-07-29

**Authors:** Jufen Liu, Gong Chen, Iris Chi, Jilei Wu, Lijun Pei, Xinming Song, Lei Zhang, Lihua Pang, Youli Han, Xiaoying Zheng

**Affiliations:** 1 Institute of Population Research, Peking University, Beijing, China; 2 Institute of Reproductive and Child Health, Peking University Health Science Center, Beijing, China; 3 School of Social Work, University of Southern California, CA, USA; 4 School of Health Administration and Education, Capital Medical University, Beijing, China

## Abstract

**Background:**

Considerable socioeconomic and health inequalities have been reported in China. However, because of a lack of appropriate data, limited research has been conducted on variations in disability-free life expectancy (DFLE) among older adults. This study aimed to use the most up-to-date disability survey data to explore geographical variations in DFLE at age 60 in China and to identify the socioeconomic and health care factors that partially account for these variations.

**Methods:**

This study used 2006 mortality data extrapolated from the 1990 and 2000 Census and disability data from a national disability survey conducted in 2006. Disability was performance based and was diagnosed by trained physicians. DFLE was calculated by region using the Sullivan method. Multiple linear regression models by gender were conducted to explore correlates of DFLE.

**Results:**

DFLE at age 60 varied widely by region, from 11.2 to 20.8 years in 2006. Per capita gross domestic product, proportion of urban residents, and access to health care were the primary factors associated with geographical variations in DFLE.

**Conclusion:**

The pattern of differences in DFLE by region mirrors the pattern of regional economic development in China. Countermeasures to decrease regional differences in DFLE include accelerating regional economic development and improving health care distribution.

## Background

China, the world's most populous and dynamic society, has made economic growth a priority since the second social revolution in 1978. The average 10% economic growth a year for the past three decades has produced dramatic improvements in the living standards of Chinese citizens. Meanwhile, life expectancy at birth has increased markedly from 65.3 years in 1975-1980 to 73 years in 2005-2010 [1]. Despite major gains in total life expectancy (TLE), there are considerable social and health inequalities and poor access to heath care in rural and less developed areas of the country [2]. The National Research Council's Panel on Urban Population Dynamics found that modern-day urban populations live longer than rural populations and that there is wide intra-region variation in life expectancy [3]. The results of a recent study examining late-life health discrepancies in Beijing indicated that urban residents have an advantage in terms of healthy life expectancy mainly because of advantages in socioeconomic status and access to health services [4]. In addition to the urban/rural gap, studies show that substantial regional economic disparities still exist [5] in education and health care [6]. As the country with the world's largest older population, China is aging at an extraordinarily rapid rate: The percentage of its population aged 60 or older increased from 7.4 in 1980 to 12.3 in 2010 [1]. It is the significant differences in economic and social life between urban and rural China that appear to be negatively influencing the chances of survival of older adults in rural areas [7]. Given these economic disparities and the rapidly aging population, research on regional differences in health status among Chinese older adults is warranted.

A frequently used indicator of health, life expectancy measures length of life without considering the health-related quality of that life. Disability-free life expectancy (DFLE), a combined measure of mortality and disability, is the average number of years spent free of disability by a person of a particular age. DFLE is a meaningful indicator of health at the population level.

Although a handful of studies have examined the TLE and DFLE of older adults in China, gaps in knowledge remain. For example, some studies on DFLE focused only on one city or province [8,9], some reported data more than 20 years old [10,11], and others studied only the oldest old [12]. Our previous research examined the trend in DFLE over the past two decades [13], and one recent study examined only urban/rural differences in healthy life expectancy [4]. No study has yet to examine regional differences in DFLE. Yet monitoring regional differences in DFLE will provide information about extent to which the Chinese health care system is achieving its aim of equal access for all.

Until now only part of the complex mechanisms leading to regional disparities in health have been explored (e.g., economics [4,14], social factors [15], lifestyle [16], and access to health services [4]). Bajekal examined variations in healthy life expectancy in England between 1994 and 1999 and found that both life expectancy and healthy life expectancy improved considerably across affluence deciles [14]. Bone et al. used multiple regression analysis with a range of factors for 115 locales in England and Wales to show that low healthy life expectancy was linked to areas of low social class, high employment, and low population density [15]. Similar results have been found for Canada [17]. An ecological analysis of Spain revealed that DFLE was related to social conditions (illiteracy, unemployment) and lifestyle factors (smoking). Weak relations were found with health care supply [16]. In The Netherlands, healthy life expectancy was related to socioeconomic indicators of the region (average income, percentage of the population that was unemployed, and percentage of the population with low education), lifestyle indicators (percentage of smokers and percentage of heavy drinkers), and health care supply indicators (number of hospital beds and number of general practitioners per 1, 000 population) [18]. Only social conditions and lifestyle differences between regions were negatively associated with healthy life expectancy, and health care supply variables showed no clear relationship. Zimmer et al. examined the effect of the number of medical facilities in the Chinese community on mortality but found none, likely because number of facilities may not translate well into quality of service or capacity of care. Lack of appropriate measures such as number of doctors, nurses, or available beds have prevented these researchers from conducting further analysis [7]. However, the present study, which is based on data on health care services at the regional level, will fill this research gap. Moreover, just as life expectancy varies between men and women, so does DFLE [13]. An unanswered question is whether the correlates of DFLE also vary between men and women.

In order to address the gaps in knowledge, we analyzed geographical variations in DFLE in China using the most up-to-date data on clinician-diagnosed disability. We also identified the socioeconomic factors associated with regional variations for both sexes in these data.

## Method

We used morbidity and mortality data to calculate DFLE for 31 administrative divisions of China: 22 provinces, 5 autonomous regions, and 4 direct-controlled municipalities (i.e., Beijing, Tianjin, Shanghai, and Chongqing). The autonomous regions and direct-controlled municipalities have equal status with the provinces. A description of each data set and the method used to calculate DFLE follows (also see Liu et al. [13] for more details).

### Morbidity Data

The prevalence data used to calculate DFLE came from China National Sample Survey on Disability conducted in 2006, which comprises the most up-to-date, detailed, and nationally representative data on disability. Utilizing stratified, multi-phase, and cluster probability sampling designs, the study covered all provinces, autonomous regions, and direct-controlled municipalities in mainland China, allowing researchers to analyze disability by region. Results of a post-survey quality check showed omission rates of 1.31 per thousand in the resident population and 1.12 per thousand in the disabled population. The accuracy of the data was greater than 95%. The data were nationally representative and reliable [19]. The study was approved by the Ethics Committee of the China Disabled Persons' Federation.

The survey was designed according to the *International Classification of Functioning, Disability and Health *[20]. In line with China's current disability standards, the present study defined a disabled person as someone suffering from one or more abnormalities in anatomical structure or the loss of a certain organ or function (either psychological or physiological) who has lost (totally or in part) the ability to perform an activity in the normal way [13]. The study considered visual, hearing, speech, physical, intellectual, and mental disabilities. For the purposes of the present research, people with one or more of the aforementioned disabilities were regarded as disabled. During data collection, interviewers screened respondents with the following questions: Do you have any problem with your eyes/ears/speech/physical activity/intellectual activity/mental activity? People who scored positive for potential problems were suspected of being disabled and were taken to a physician for further screening. Using professional tools to check body function and structure, activity, and participation, trained physicians gave the final diagnosis and assessment of disability. Hearing disability referred to a unilateral hearing loss >40 Db HL (in the better ear) as tested with in-ear headphones; intellectual disability referred to an intelligence quotient <70 as tested with the Wechsler Intelligence Scale for Adults-Chinese Revised short forms and adaptive behavioral disorder. Hence, diagnoses of disability in the present study were performance based instead of self-reported, as is common in other studies. A total of 85, 260 persons from the subsample of 354, 859 adults aged 60 and older in 2006 were classified as disabled.

### Mortality Data

Our previous report [13] used mortality data estimated by the United Nations Population Division, but these data were for the whole country. Mortality data by administrative division are not available. Thus, mortality by age and gender for each administrative division was derived from 1990 and 2000 census data. We assumed that age-specific mortality, m(x,t), follows an exponential function, and the natural logarithm of age-specific mortality, ln[m(x,t)], follows a linear function [21]. Given the absence of very rapid changes in mortality (due to unusual circumstances such as wars, disaster, etc.), we assumed that the annual change in mortality rate was consistent and the change by age structure was stable over the two decades. Based on this assumption, we used the extrapolation method to calculate 2006 mortality data according to these divisions.

### Covariates

Explanatory variables were grouped into two categories, socioeconomic indicators and health care indicators, both at the level of the administrative division. Per capita gross domestic product (GDP) was calculated by dividing the total GDP by the population of the administrative division. Proportion of urban residents refers to the urban population (the people residing in urban residential communities) as a percentage of the total population. Illiteracy rate refers to the percentage of people aged 60 and older who could not read or write or who knew fewer than 1500 Chinese characters. The proportion of house utilities is the number of houses that had modern utilities (e.g., running water, shower, toilet, gas or electricity as fuel) divided by the total number of houses. Health care indicators include the number of hospital beds and number of clinicians and nurses, both of which were measured per 10, 000 residents. Illiteracy rate and house utilities came from the *China 1% National Population Sample Survey in 2005 *[22], and all other indicators came from the *China Statistical Yearbook 2007 *[23].

### Method

We used the Sullivan method to calculate DFLE for each administrative division to determine the average number of years spent free of disability at age 60. The Sullivan method uses period life tables and the prevalence of disability in each age group to divide the number of person-years into years with and without disability [24]. Estimates of DFLE at age x were obtained by summing the number of years lived without disability over all age groups and dividing this by the size of the life table cohort at age x. The Sullivan method is the most widely used method for calculating DFLE, as it uses more readily available data and simpler assumptions than the multistate life table method [25].

The correlation between socioeconomic factors and variation in DFLE was explored using multiple linear regression models.

## Results

### Prevalence of Disability

The distribution of disability varied significantly by region. The prevalence of disability was higher in western (14.9%) and middle (13.5%) regions than in eastern regions (10.3%), which had the lowest prevalence of disability.

### Life Expectancy and DFLE

**Figure 1:**
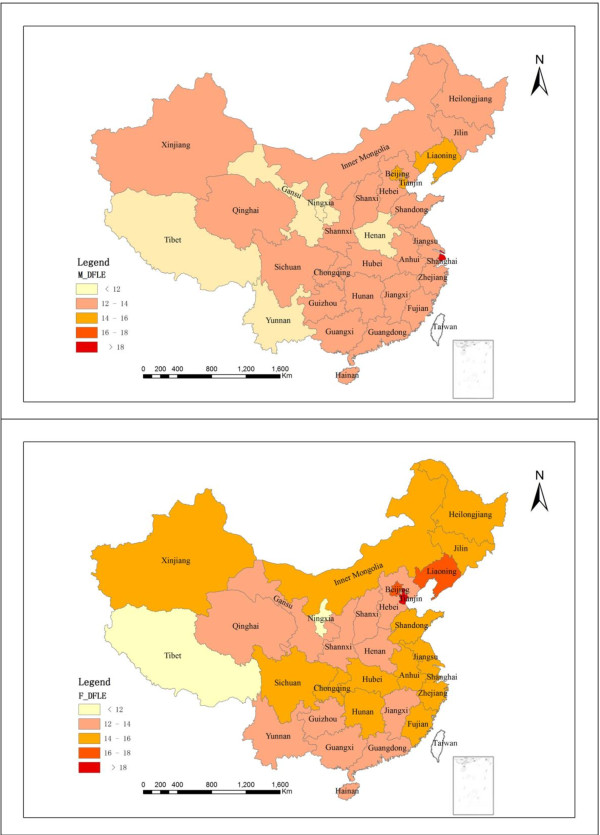
**Disability-Free Life Expectancy (DFLE) at age 60 for men and women, by Administrative Division**. Figure 1 depicts DFLE at age 60 by administrative division separately for men and women. Substantial disparities in DFLE exist among the 31 provinces. Generally speaking, DFLE is highest in eastern areas, followed by middle areas and western areas.

Figure 1 shows DFLE at age 60 among all administrative divisions, separated by gender. Both life expectancy and DFLE showed clear regional differences. Eastern regions had highest life expectancy and DFLE, whereas western regions had the lowest life expectancy and DFLE (see also Table 1).

**Table 1:** Prevalence of Disability and TLE, and DFLE at Age 60, by Administrative Division and Region, 2006(%)

Province	Prevalence (%)	TLE	DFLE	Standard Error of DFLE	Proportion (%) (DFLE/TLE)
**Total**	**12.7**	**18.8**	**13.9**	**0.01**	**73.5**

Beijing	10.5	21.5	15.9	0.09	74.1
Tianjin	8.6	21.8	16.4	0.10	75.1
Hebei	14.5	18.5	12.8	0.07	69.1
Liaoning	10.3	19.7	15.6	0.08	79.5
Shanghai	6.2	26.7	20.8	0.11	78.1
Jiangsu	9.3	19.2	14.4	0.06	75.4
Zhejiang	9.8	18.7	14.2	0.06	76.0
Shandong	12.4	19.5	13.9	0.06	71.4
Guangdong	11.0	18.2	13.2	0.06	72.4
Guangxi	12.1	18.3	12.4	0.07	67.8
Hainan	11.0	18.0	13.1	0.10	72.5
Fujian	7.4	18.8	14.3	0.17	76.3

**Eastern **region	**10.3**	**19.9**	**14.8**	**0.09**	**74.0**

Shanxi	14.0	18.6	13.4	0.09	72.2
Inner Mongolia	16.1	17.7	13.5	0.09	76.5
Jilin	14.4	18.5	13.3	0.09	71.9
Heilongjiang	12.4	18.7	14.6	0.09	77.8
Anhui	10.7	18.2	14.0	0.06	77.1
Jiangxi	12.2	18.3	13.5	0.08	73.8
Henan	17.1	18.0	12.2	0.06	67.9
Hubei	13.2	18.1	13.9	0.07	76.8
Hunan	11.4	18.3	14.4	0.06	78.5

**Middle **region	**13.5**	**18.3**	**13.6**	**0.08**	**74.7**

Chongqing	10.1	17.8	14.6	0.06	81.9
Sichuan	12.7	18.3	13.7	0.06	75.2
Guizhou	10.5	18.1	13.0	0.08	71.6
Yunnan	17.4	17.8	12.2	0.08	68.5
Tibet	17.6	16.4	11.4	0.16	70.0
Shaanxi	14.7	18.3	13.2	0.09	72.0
Gansu	18.8	18.1	11.9	0.11	65.7
Qinghai	15.7	17.7	12.6	0.15	71.2
Ningxia	19.6	18.0	11.2	0.14	61.9
Xinjiang	11.7	18.2	14.5	0.11	79.4

**Western **region	**14.9**	**17.9**	**12.8**	**0.10**	**71.7**

Note: Prevalence: Proportion of people with disability; TLE: Total life expectancy; DFLE: Disability-free life expectancy; Proportion: Proportion of DFLE to TLE.

Because the economy is more developed in the four direct-controlled municipalities (i.e., Beijing, Tianjin, Shanghai, and Chongqing) than in the other administrative divisions, we ran a separate analysis excluding these municipalities. Results showed a reduction in both life expectancy and DFLE; the trend from east to west remained clearly visible. That is, DFLE decreased from 14.8 to 13.8 years in the east and from 12.8 to 12.6 years in the west.

DFLE for both sexes was estimated at 13.9 years in 2006. This figure masks considerable variations in DFLE by region: from 11.2 years in Ningxia to 20.8 years in Shanghai, a gap of close to 10 years. A discrepancy of this magnitude suggests that improvements in health are possible in the regions where DFLE estimates are lowest. Although women had a greater TLE, the proportion of DFLE to TLE was smaller for women than men. Women in the eastern and western regions had a lower proportion of DFLE to TLE, whereas those in the middle region had a small advantage in this regard.

We categorized the provinces into groups based on life expectancy and DFLE. Shanghai led with an estimated average life expectancy at 60 of 26.7 years and a DFLE of 20.8 years. Tibet had the lowest life expectancy and DFLE which is 16.4 and 11.4 respectively. At the same time, the proportion of DFLE to TLE shows different pattern with life expectancy and DFLE. Higher life expectancy does not necessarily mean a greater proportion of DFLE. For example, Shanghai had the highest life expectancy but not the greatest proportion of DFLE to TLE. Chongqing had the greatest proportion of DFLE to TLE, but its overall life expectancy fell somewhere in the middle. This indicates that researchers should take caution when using a single indicator to interpret the health status of a population. The distribution of DFLE was very similar to trends in economic development in the eastern and western regions.

### Regression of Factors Explaining Variation in DFLE

Table 2 shows the correlation between socioeconomic indicators and health care indicators and variation in DFLE. There were significant regional differences in both life expectancy and DFLE (*P *< 0.01). The other significant correlates were per capita GDP, proportion of urban residents, illiteracy rate (60+), fewer household utilities, and health care resources (*P *< 0.01).

**Table 2:** Correlations of Life Expectancy and Disability-Free Life Expectancy at Age 60, 2006

Variables	Life Expectancy		Disability-free Life Expectancy	
	
	Pearson correlation	P Value	Pearson correlation	P Value
Life Expectancy	1	.	0.89^†^	0.000
Disability-free Life Expectancy	0.89^†^	0.000	1	.
Per Capital GDP	0.86^†^	0.000	0.79^†^	0.000
Proportion of Urban Residents	0.81^†^	0.000	0.79^†^	0.000
Illiteracy rate (60+)	-0.59^†^	0.000	-0.62^†^	0.000
Proportion of No Shower Facility at House	-0.53^†^	0.002	-0.53^†^	0.002
Proportion of No Toilet at House	-0.24	0.190	-0.18	0.325
Proportion of No Water at House	-0.63^†^	0.000	-0.59^†^	0.001
Proportion of No Gas and Electricity as fuel at House	-0.76^†^	0.000	-0.73^†^	0.000
Hospital beds Per 10, 000 Residents	0.72^†^	0.000	0.69^†^	0.000
Clinicians and Nurses Per 10, 000 Residents	0.66^†^	0.000	0.61^†^	0.000

Note: N = 31 administrative divisions;^† ^*P *< 0.01

The multiple linear regression results showed that proportion of urban residents and numbers of hospital beds per 10, 000 people were the significant factors explaining regional differences in DFLE among women (see Table 3). These factors accounted for 40% of the variation. Among men, per capita GDP and numbers of hospital beds per 10, 000 people accounted for around 28% of the variation.

**Table 3:** Multiple Regression of DFLE at Age 60 (Backward Selection, Standardized Coefficient), 2006

Variables	Men	Women
Per Capital GDP	0.53^†^	
Proportion of Urban Residents		0.68^†^
Illiteracy rate (60+)		
Proportion of No Shower Facility at House		
Proportion of No Water at House		
Proportion of No Gas and Electricity as fuel at House		
Hospital beds Per 10, 000 Residents	0.63^^^	0.87^†^
Clinicians and Nurses Per 10, 000 Residents		
Adjusted R^2^	0.29	0.41

Note: N = 31 administrative divisions;^† ^*P*<0.01,^^^*P*<0.05

## Discussion

Although numerous health expectancy studies have been conducted in the United States and Europe, few such studies have been conducted in developing countries. In one study, researchers compared age and gender patterns of health expectancy among older adults in six Asian settings (China, Indonesia, the Philippines, Singapore, Taiwan, and Thailand) [26]. The health indicator used was self-assessed health, a subjective measure of health that can vary across cultures. A recent study showed that rural populations have a pronounced disadvantage in terms of survival, and there are large differences in life expectancy between provinces in China [27]. Describing and addressing health inequalities among older adults are key focuses of public health agendas in China. The current paper is the first to show regional variations in DFLE among older adults in China.

Our results confirm that considerable differences in healthy life expectancy exist across China. Studies from the United Kingdom have shown the existence of regional differences in impairment and poor health at older ages. Moreover, differences in health expectancy are greater between regions than are differences in life expectancy [28]. Despite major gains in life expectancy and improved health and living standards, evidence remains of considerable social and health inequalities and poor access to health care in less developed areas, such as in western regions. Hence, health and life expectancy improvements have typically been greater in the more developed eastern and northern areas of China.

With the steady decline in mortality in China, life expectancy has steadily increased in every administrative division. Disability due to physical, mental, or emotional health problems is a major public health issue, resulting in the reduction of life quality and increased dependence on the health care system [29]. Although the prevalence of disability has increased in most administrative divisions as well as in the country as a whole [13], DFLE and the proportion of DFLE to TLE vary. Regional differences in DFLE mirror regional economic differences. Variations in socioeconomic development and natural environment may contribute to this variation [30].

The National Bureau of Statistics of China has divided the country into three economic zones based on economy and geography. The eastern region consists mainly of more developed coastal provinces; the western region is less developed and has a poorer economy. The economy of the middle region, lying in the inner mainland, falls somewhere between that of the western and eastern regions. The results of the present research, which show that higher life expectancy and DFLE accompany higher economic status, are in line with the results of other studies [31].

Socioeconomic development, urbanization, and health care resources are major factors that explain regional disparities in DFLE among older adults in China. A decrease in healthy life expectancy of older people was associated with lower socioeconomic conditions, which is corresponding with finding from Japan [32]. Although the root causes of health inequalities include a very complex array of factors, lack of access to effective health care is one crucial factor. Investments in infrastructure, health care, and economic development in rural areas would benefit rural elders [7]. The policy implication is to strengthen investment in rural China and in western China to reduce health inequalities. The government should pay more attention not only to economic development but also to inequalities in the economy as well as health care distribution between regions.

The factors explaining variation in DFLE vary little between men and women. Socioeconomic factors and health care resources play important roles in variations in DFLE. However, unlike other studies, our results did not show a significant role of education for either men or women. One possible reason is the homogeneous nature of education among Chinese older adults, but more detailed analysis is needed to confirm this.

There are limitations to this study. First, this study was cross-sectional in nature, meaning it offers limited opportunities for exploring the causal relationship between socioeconomic factors and DFLE. Second, although the Sullivan method is a popular method of calculating DFLE, it has several drawbacks, such as the fact that its assumptions constrain the portrayal of the expected life cycle or the functional status histories of persons who are exposed to current mortality and morbidity conditions. Furthermore, it does not allow for individual recovery from the original state to another state [33]. Hence, DFLE may have been underestimated. Moreover, the different definition and measurement of disability used here prevent comparisons with data from other countries.

## Conclusion

Despite its limitations, this study expands the current literature in several ways. Using the most up-to-date, nationally representative, performance-based data on disability, this article provides the first estimates of regional variations in DFLE across China and possible factors explaining this variation. Despite increases in life expectancy and DFLE, substantial disparities by administrative division still exist in healthy life expectancy, mirroring patterns in regional economic development. Accelerating economic development and urbanization and improving the distribution of health care resources in disadvantaged regions will help reduce health disparities in China.

## Competing interests

The authors declare that they have no competing interests.

## Authors' contributions

JFL initiated the study, analyzed data and wrote the original article. GC participated in study design and data analysis. He contributed equally to this work with JFL. IC contributed to manuscript writing and language editing. JLW participated in organizing and managing the data, drawing the figure. LJP contributed to the design of the study. XMS provided critical advices on writing and revising the article. LZ participated in gathering the data and data analyses. LHP and YLH contributed to writing and modifying the paper. XYZ designed the study and managed the study throughout the work. All authors read and approved the final manuscript.

## Pre-publication history

The pre-publication history for this paper can be accessed here:

http://www.biomedcentral.com/1471-2458/10/446/prepub
